# A multi-phase reactions for organic carbon removal in bio-electrochemical reactors employing Fe_2_O_3_-synthesized nanoparticles

**DOI:** 10.1371/journal.pone.0355332

**Published:** 2026-08-20

**Authors:** Samaneh Abolli, Kazem Naddafi, Kamyar Yaghmaeian, Vahide Oskoei, Mahmood Alimohammadi

**Affiliations:** 1 Department of Environmental Health Engineering, School of Public Health, Tehran University of Medical Sciences, Tehran, Iran; 2 Center for Air Pollution Research (CAPR), Institute for Environmental Research (IER), Tehran University of Medical Sciences, Tehran, Iran; 3 Center for Solid Waste Research, Institute for Environmental Research, Tehran University of Medical Sciences, Tehran, Iran; 4 School of Life and Environmental Science, Deakin University, Australia; 5 Center for Water Quality Research (CWQR), Institute for Environmental Research (IER), Tehran University of Medical Sciences, Tehran, Iran; 6 Health Equity Research Centre (HERC), Tehran University of Medical Sciences, Tehran, Iran; Linköping University: Linkopings universitet, SWEDEN

## Abstract

Two Poly(methyl methacrylate) reactors were fabricated, and the flow of synthetic Dissolved Organic Carbon (DOC) was maintained. Humic acid was used as the source of organic carbon, with a water pH of 7–8.5, TDS of 220–240 mg/L, and EC of 0.4–0.5 mS/cm. Iron nano-particles were synthesized via the sol-gel method and characterized by Energy-Dispersive Spectroscopy (EDS), Dynamic Light Scattering (DLS), Fourier Transform Infrared Spectroscopy (FT-IR), and Field Emission Scanning Electron Microscopy (FE-SEM). The reactors were operated sequentially in three distinct phases: physicochemicals, biological, and bio-electrochemicals, with a direct current applied under flow-through conditions. The biological samples were stained with acridine orange (AO) and examined using CLSM. The hydraulic retention time (HRT) of each column was approximately 293 min (4.9 h), the effective working volume was 440 mL and the operating flow rate was 1.5 mL min ⁻ ¹. The Fe-FAC reactor demonstrated superior DOC removal, reducing concentrations from 3.61 ± 1.68 mg/L to 1.64 ± 1.34 mg/L, compared to the GAC column’s reduction (4.26 ± 3.21 mg/L to 2.56 ± 1.08 mg/L). Flow cytometry (FCM) analysis revealed that Fe–GAC exhibited a lower event density than GAC at comparable dilution levels, suggesting a regulatory effect on microbial populations and biofilm development. In Phase I, both reactors demonstrated high DOC removal efficiencies. During phase II, removal efficiency declined in both systems. By Phase III, GAC exhibited a marked decline in performance, accompanied by the release of DOC, while Fe-GAC maintained a superior and more stable removal efficiency.

## 1. Introduction

Dissolved Organic Carbon (DOC) is a key indicator of water quality, playing a crucial role in the assessment and management of water treatment and wastewater processes. It represents the fraction of organic matter in water that can pass through a filter with a pore size of 0.45 µm. DOC plays a vital role in environmental and health-related processes, serving as a key indicator of organic matter in water bodies [1–3]. High concentrations of DOC can lead to aesthetic problems, such as unpleasant taste and odor, and promote bacterial regrowth in distribution systems. Therefore, monitoring DOC is essential for maintaining the overall quality of water [4,5].

In drinking water treatment, elevated DOC levels can react with disinfectants such as chlorine, leading to the formation of harmful disinfection byproducts (DBP) [6–8]. Consequently, controlling and minimizing DOC is crucial to reducing the formation of these harmful compounds. Additionally, the presence of DOC can adversely affect the efficiency of various water treatment processes. It affects coagulation efficiency, increases the chemical demand for disinfection, and can lead to membrane fouling during the filtration process. Therefore, effective management of DOC is essential for maximizing water treatment performance and ensuring the safety of drinking water [9,10].

The properties of GAC media play a crucial role in the effective removal of DOC and natural organic matter (NOM), which are recognized as primary precursors to the formation of DBP in drinking water treatment. Empirical studies demonstrate that the adsorption performance and overall DOC removal efficiency of GAC are intrinsically linked to its physicochemicals attributes, including pore size distribution, specific surface area, and particle size [11]. Beyond its absorptive capacity, GAC supports the development of biofilms that enhance the Biodegradation of NOM, thereby augmenting DOC removal through combined physicochemicals and biological mechanisms over operational timescales [3,12–14].

The doping of iron nano-particles on GAC is highly significant due to the synergistic enhancement it provides for water treatment applications. Iron nano-particles, particularly zero-valent iron encapsulated within a carbonaceous matrix, markedly improve the adsorbent’s stability, reactivity, and overall performance by preventing nano-particle aggregation and preserving high surface reactivity. Encapsulation on GAC develops a me-soporous architecture characterized by a high surface area and hydrophobicity, which enhances the rapid and efficient adsorption of organic contaminants from water. Moreover, the iron core facilitates magnetic separation and catalyzes heterogeneous Fenton oxidation, enabling efficient regeneration and repeated reuse of the adsorbent [15–18].

**Fig 1:**
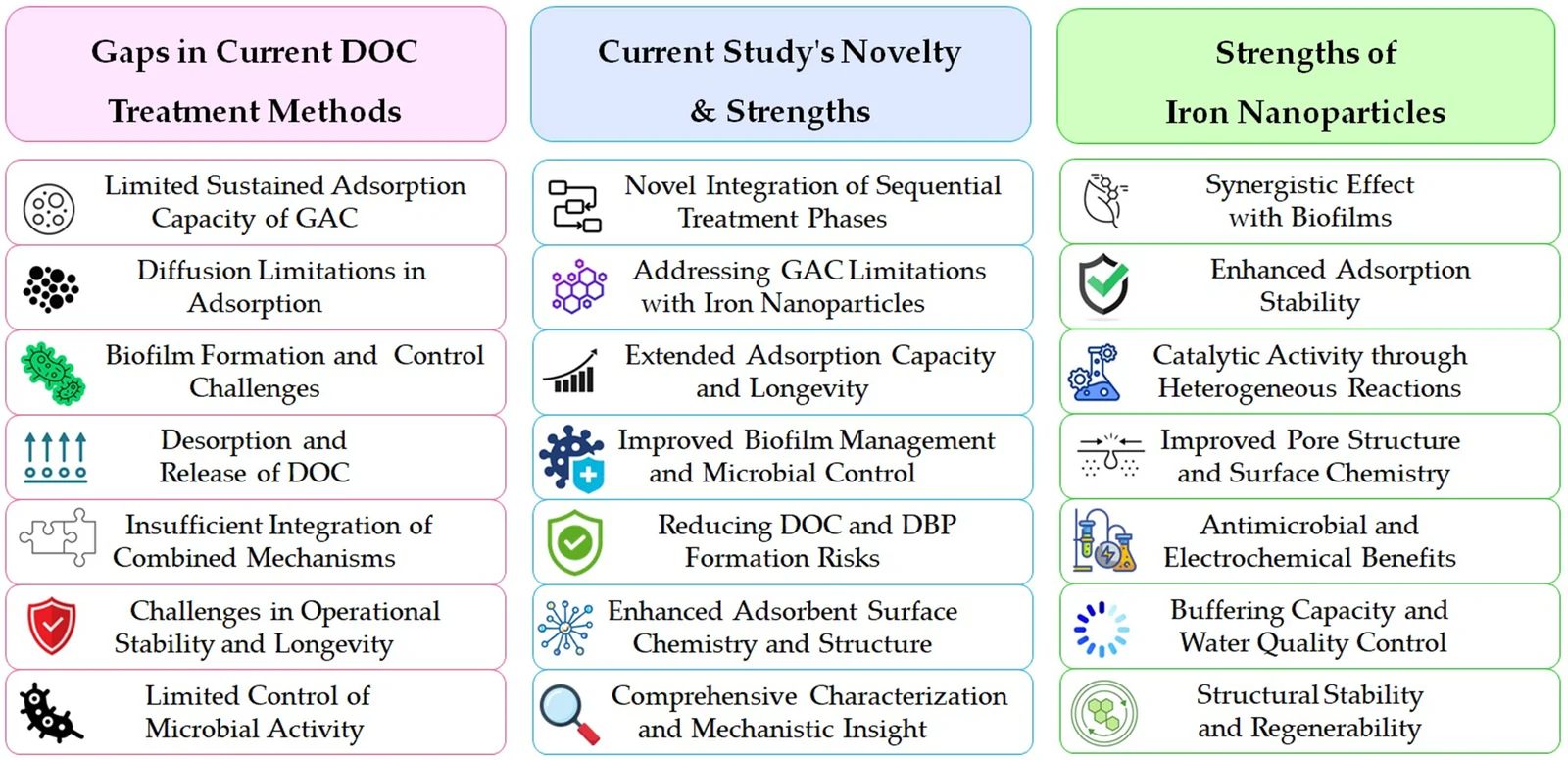
Conceptual framework of the study.

To address the limitations of raw GAC, this study explores the innovative application of a Fe-GAC composite as a more efficient approach for the removal of organic pollutants, as illustrated in Fig 1. Therefore, the main objectives of this study were to: **a)** compare the adsorption capacity of an Fe-GAC composite with that of GAC across three operational phases, **b)** assess the contribution of each phase to DOC adsorption, and **c)** estimate the biomass attached to the adsorbents as an indicator of Biodegradation within the reactors. The novelty of this study resides in the integration of the treatment stages under continuous-flow operation and the extended monitoring of biofilm behavior and reactor performance over time.

**Chart 1:**
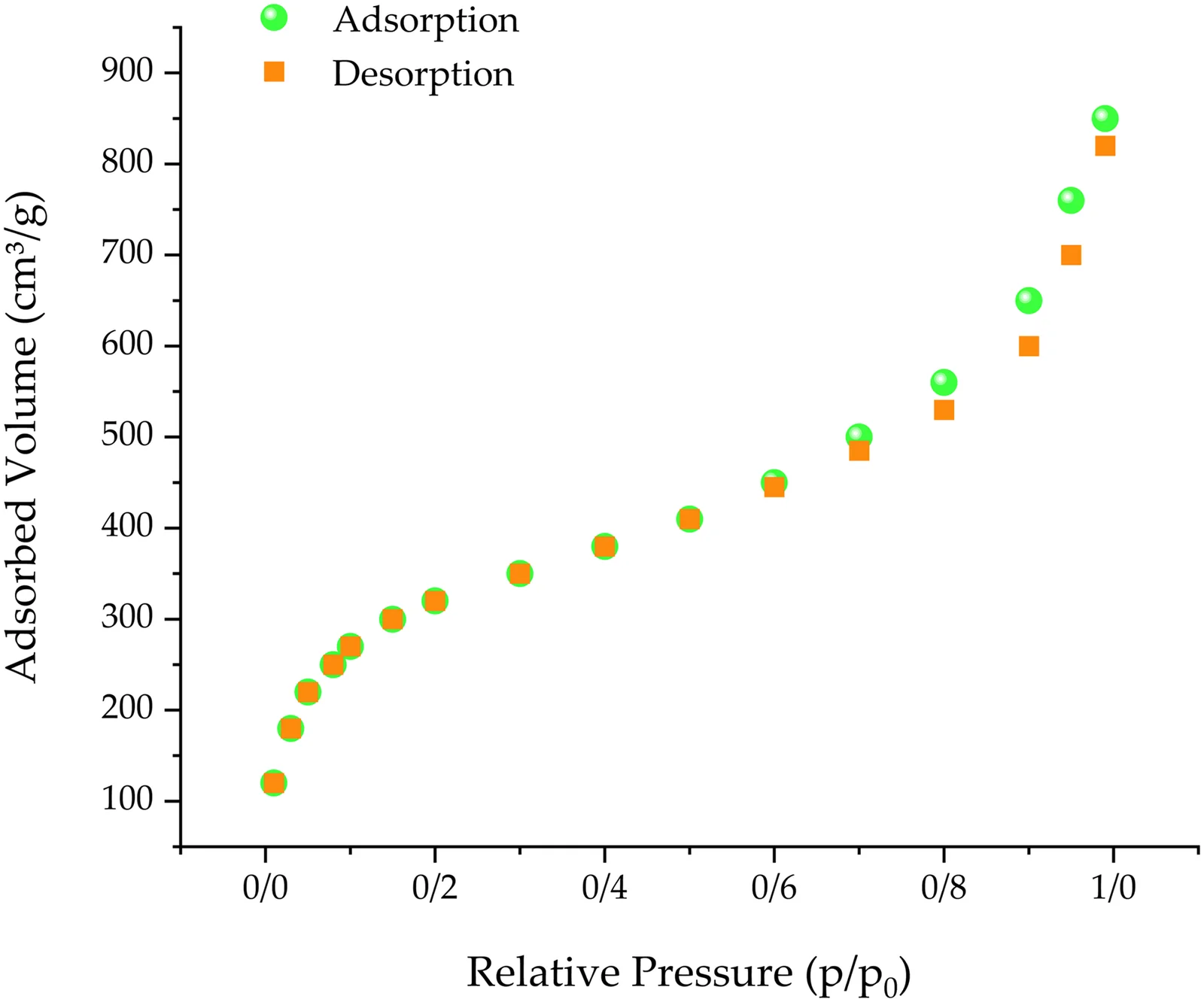
Nitrogen adsorption–desorption isotherm of the GAC.

## 2. Materials and methods

The study was structured into three main components: system design and construction, nano-particle synthesis, and operational/testing evaluation. The operational phase was further divided into three sequential stages: physicochemicals, biological, and bio-electrochemicals phases.

### 2.1 Reactor configuration

**Fig 2:**
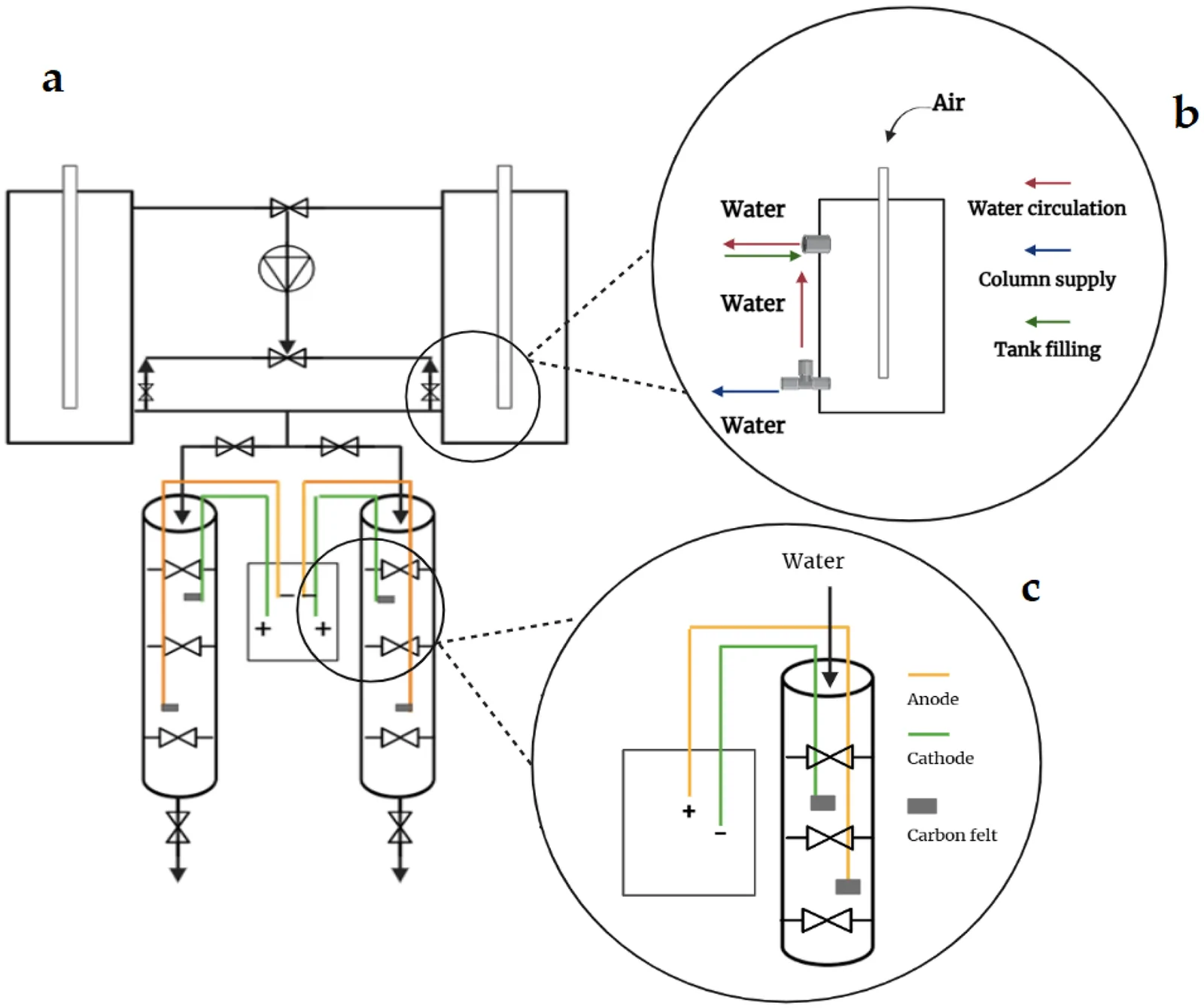
Configuration of the continuous-flow column system: (a) overall reactor setup, (b) influent tank design with controlled aeration and mixing, and (c) electrode arrangement for bioelectrochemical operation.

Two columns, each with an active volume of 440 mL, were constructed from poly(methyl methacrylate). Each column was segmented at 5, 21, and 37 cm from the base, with a stainless-steel valve installed at each level. The lower end of each column was packed with stainless-steel mesh (mesh 304) and sand (Uniformity Coefficient = 1.98) to prevent the escape of activated carbon granules. Three-layer polyethylene tanks were employed to supply the required flow of synthetic DOC solution. To avoid the effect of flow height on pressure and flow rate, the tank lid was completely sealed, and a pipe was used to transfer air into the tank. The mixture within the tanks was constantly stirred, and once the volume of the first tank was depleted, flow from the second tank was directed into the system. Fig 2 shows the reactor design and its operational configuration.

**Chart 2:**
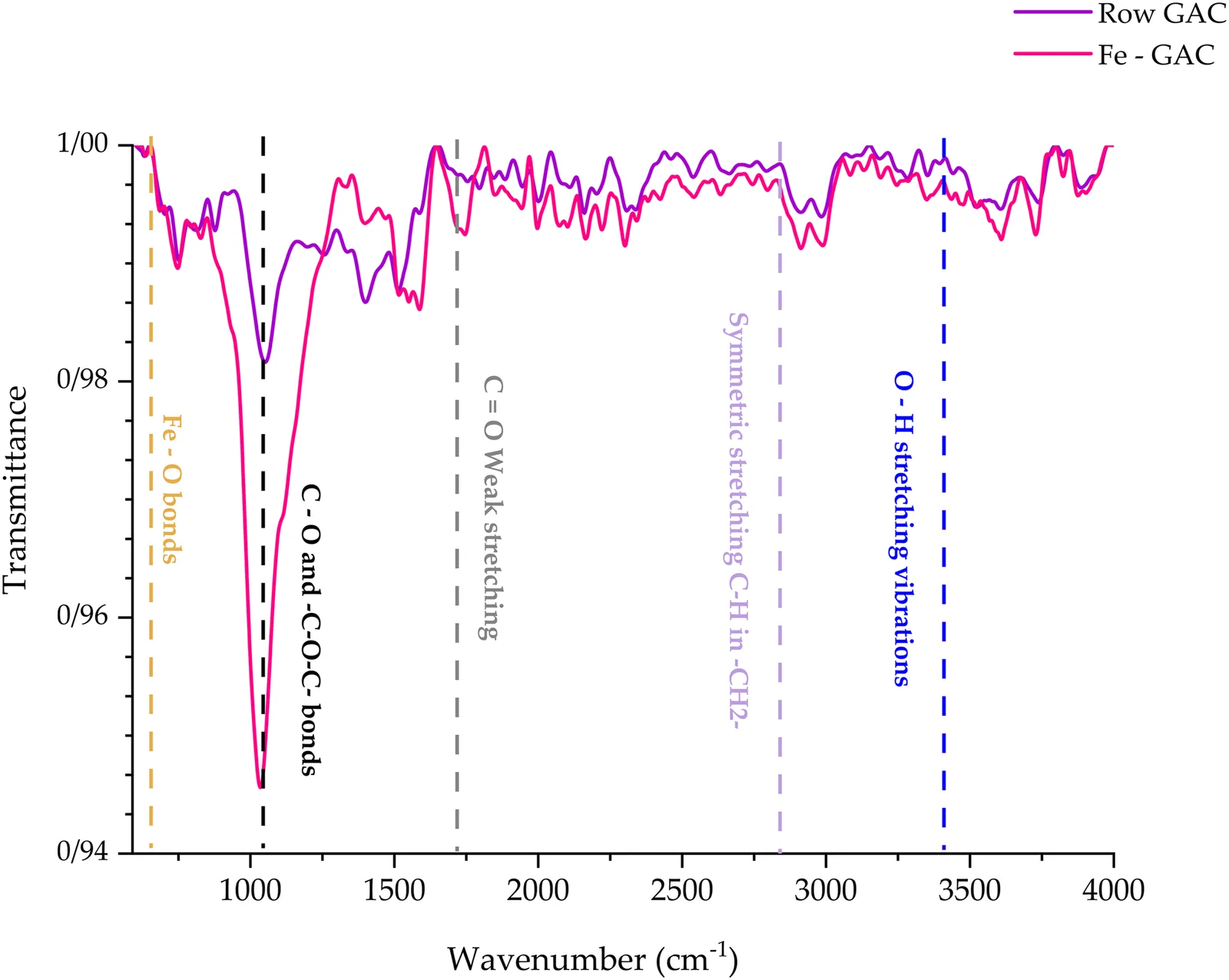
FT–IR spectra of raw GAC and Fe–GAC.

### 2.2 Synthesis of Fe–GAC

Iron nano-particles were synthesized using the sol-gel method [19]. Initially, 20 g of Jacobi granular activated carbon was mixed with 900 ml of distilled water. To prepare a 3 wt% Fe₂O₃ solution, 0.6 g of ferric chloride (FeCl₃·6H₂O) was dissolved in 100 mL of distilled water, followed by the addition of 46 μL of ammonium hydroxide (NH₄OH, 14.69 M) to achieve a hydroxide-to-iron ion ratio of 3:1, resulting in the formation of iron hydroxide (Fe(OH)₃). The iron hydroxide solution was gradually introduced to the activated carbon granules in a drop-wise manner. The mixture was then stirred at 450 rpm for 24 hours, after which it was subjected to ultrasonic treatment (Model: D-78224 Singen/Htw, Type: TI-H-5) at a frequency of 35 kHz for 30 minutes. The mixture was subsequently filtered using a vacuum pump (Model: HP1400V, 120 L/min, 50 Hz, −700 mmHg) in combination with a filter unit (F2040-125, chm, 125 mm, Spain). Finally, the granules were left at room temperature for 24 hours to dry. Subsequently, the Fe-GAC was subjected to glacial acetic acid vapor under controlled pressure at 80 °C for 120 minutes. Subsequently, the material was reheated at 80 °C for 30 minutes to eliminate any residual acetic acid. In the calcination step, the mixture was exposed to air inside the furnace at 400 ^◦^C for 120 minutes. An air pump with a flow rate of 16 LPM was used to supply air into the furnace. Finally, Fe-GAC was sieved using a 100 µm mesh to achieve a uniform grain size before being loaded into the columns. The morphological properties and chemical composition of Fe_2_O_3_ nano-particles were assessed with Energy Dispersive Spectroscopy (EDS, Model: EDS SAMX – France), and the size distribution of iron nanoparticles was analyzed using Dynamic Light Scattering (DLS) with a SZ-100z Dynamic Light Scattering and Zeta Potential Analyzer from Horiba Jobin Yvon. Field Emission Scanning Electron Microscopes (FE-SEM, model: Mira 3-XMU equipped with LVSTD detector) tests, and the FE-SEM test was repeated after 4 months for GAC and Fe-GAC. Fourier-transform infrared spectroscopy (FT–IR, model: Tensor 27 & Equinox 55, Bruker – USA) was used to identify and characterize materials by measuring the infrared absorption spectra. The characteristics of the GAC that was used, based on the Brunner-Emmett-Teller (BET, Model: micrometeoritic – Micro-active II Plus) analysis at a temperature of −196.904 ^◦^C, with 11.4019 cm³ of free space in the presence of inert nitrogen gas.

### 2.3 Operational phases

The reactors were operated sequentially in three phases:

- Phase I: Physicochemicals (adsorption-dominated)
- Phase II: Biological (biofilm development with nutrient addition)
- Phase III: Bioelectrochemical (application of direct current)

Natural organic matter (NOM), a key precursor in the formation of THMs, can be generally categorized into two groups: humic substances, including fulvic and humic acids, and non-humic substances, such as carbohydrates, hydrocarbons, lipids, and amino acids. Due to its complex and variable nature, NOM is usually quantified using surrogate measures like TOC, DOC, ultraviolet absorbance at 254 nm, or specific ultraviolet absorbance (SUVA) [20]. Therefore, in this study, humic acid was used as a natural organic source, while DOC and UV254 nm were employed to quantify the amounts of organic matter and its reactivity.

### 3.1. Physicochemical phase

In-fluent and effluent parameters, including DOC, pH, TDS, and EC, were alternately monitored for each column. To quantify DOC, 20 mL of the effluent was passed through a 0.22 µm sterile syringe filter (Part Number SFCA030022S, Lot No. 329021174). The absorbance of the filtered sample was then measured at 254 nm using a spectrophotometer (Model: Lambda 25, PerkinElmer). Subsequently, the same sample was then analyzed using a TOC analyzer (Model: Analytik Jena GmbH - multi-N/C 3100) to determine the amount of DOC. Distilled water was used as a blank in all subsequent chemical analysis steps. The linear regression was performed using SPSS software to estimate the relations between humic acid concentration, DOC, and UV_254_ nm absorbance. The hydraulic retention time (HRT) of each column was approximately 293 min (4.9 h), calculated from the effective working volume (440 mL) and the operating flow rate (1.5 mL min ⁻ ¹).

### 3.2. Biological phase

To provide the primary microorganisms into the system during this phases, a nutritional supplement contains 20 grams soil and essential macroelement was used. The medium composition included 7.4 g/L K_2_HPO_4_, 3 g/L KH_2_PO_4_, 0.5 g/L NaCl, 1 g/L NH_4_Cl, 0.25 g/L MgSO_4_.7H_2_O, 0.01 g/L CaCl_2_, 1 g/L C₆H₁₂O₆ and 5 ml of trace elements including 0.5 g/L ZnCl_2_, 0.3 g/L MnCl_2_.4H_2_O, 3 g/L H_3_BO_3_, 2 g/L CoCl_2_.6H_2_O, 0.1 g/L CuCl_2_.2H_2_O, 0.2 g/L NiNO_3_.6H_2_O, 0.3 g/L, and Na_2_MoO_4_.2H_2_O. The resulting solution was filtered using a vacuum pump before being injected into the columns. Additionally, 40.5 mL of Na_2_HPO_4_ 0.2 M and 9.5 mL of NaH_2_PO_4_ 0.2 M were used as a buffer solution to achieve a pH of 7.4 (https://www.sigmaaldrich.com, Buffer Reference Center). Then, the solution was introduced into the columns at a flow rate of 1.5 mL/min, and the component was injected for six days. The chemical oxygen demand (COD) of the nutrient supplement was measured several days after injection. To identify the mineral ingredient of used soil, a 500 mg sample was digested with 2 mL of HNO₃ on a hot plate, followed by slow filtration (Ø 12.5 mm - F2040- 170 µm – chm). Subsequently, 2 mL of H₂O₂ was added, the final volume was adjusted to 10 mL, and the solution was analyzed for elemental composition using Inductively Coupled Plasma Optical Emission spectroscopy (ICP-OES – Model: Spectro atcos, Germany, 30 RPM).

During the biological phase, in addition to monitoring routine physicochemicals parameters, the microbial community was evaluated through culture-based methods. Isolation Medium for Iron Bacteria (Product Number I9151, Sigma-Aldrich) and Nutrient Agar (Product Number N4019, Sigma-Aldrich) were used for organism detection. The media was prepared based on the company’s instructions. After 48 hours of incubation, bacterial colonies grown on Nutrient Agar were transferred onto slides as smears, stained using the Gram staining method, and examined under an Olympus CX31 microscope at 100 × magnification. The same original outlet samples of the reactors were examined to measure bacterial cell number by flow cytometry (FCM) in 15 seconds (Sysmex Partec, Germany). Also, the dissolved oxygen (DO) was checked alternately in all phases.

### 3.3. Bio-electrochemical phase

Following the microbial phase and the establishment of microbial growth within both column beds, an electric current of 0.7 A cm ⁻ ² was applied to the columns to investigate the influence of electrical stimulation on microbial activity and DOC removal efficiency. Two copper wires, each capable of carrying a maximum voltage of 12 V, were used to supply electrical current to the columns. The wire ends were connected to carbon felt electrodes (0.2 mm thick) with a surface area of 4 cm^2^. A direct current (DC) power generator (Model: Dazheng, PS 305 D) generated 0.7 A/cm², and the electrochemicals reactor was operated in flow-through mode [21], and the current was monitored continuously. Current density, electrode material, and electrode configuration were identified as key factors influencing reactor performance and overall treatment efficiency [22]. To observe microorganisms that grow on the composite after applying electrical current with a Confocal Laser Scanning Microscopy (CLSM, Leica model TCS SPE), two samples (0.5 g) of the adsorbents (GAC and Fe-GAC) were taken from each column and centrifuged (Universal Centrifuge Premium 20000R) for 1 hour at 10,000 rpm. Then, 20 µL of centrifuged suspension of each column was combined with 10 µL of acridine orange (AO) hemi (Zinc chloride) salt, 0.2 mg/L (CAS Number: 10127- 02- 3, Sigma-Aldrich) dye, and after 15 minutes of incubation in the dark and at room temperature, the samples were placed on a slide and observed.

### 3.4. Statical analysis and visulization

Statistical analyses were performed using IBM SPSS Statistics version 26. Descriptive statistics, including mean, standard deviation, minimum, and maximum values, were calculated to summarize the characteristics and variability of the measured parameters. Independent-samples t-tests were conducted to evaluate significant differences between the GAC and Fe–GAC composite reactors under different operational conditions. Statistical significance was considered at a confidence level of 95% (p < 0.05). Data visualization, including graphs and distribution plots, was performed using Origin-pro 2024 (Origin Lab Corporation, Northampton, MA, USA) to facilitate the interpretation and presentation of treatment performance and water quality trends throughout the experimental period.

## 4. Results and discussion

The nitrogen adsorption–desorption isotherm (Chart 1) and BET textural analysis (Table 1) revealed that the raw GAC possessed a highly developed porous structure dominated by micropore with a contribution from meso-pores. The isotherm exhibited a sharp increase in nitrogen uptake at low relative pressures (P/P₀ < 0.1), indicating extensive micropore filling, while the gradual increase in adsorption capacity at intermediate and high relative pressures, accompanied by a narrow hysteresis loop, suggested the presence of mesoporous features and capillary condensation within the pore network. These observations were consistent with the BET characterization results, which showed a high specific surface area of 1106 m² g ⁻ ¹ and a Langmuir surface area of 1350 m² g ⁻ ¹. The t-plot analysis indicated a micropore surface area of 701 m² g ⁻ ¹, accounting for approximately 63% of the total BET surface area and confirming the dominant role of microporosity in the adsorbent structure. Furthermore, the GAC exhibited a total pore volume of 0.85 cm³ g ⁻ ¹ and average pore diameters of 2.64 and 2.84 nm based on the adsorption and desorption branches, respectively, suggesting a micro–mesoporous architecture. The combination of high surface area, substantial micropore content, and adequate pore volume provides abundant active adsorption sites and favorable mass-transfer pathways, highlighting the suitability of the GAC for efficient contaminant adsorption and removal.

**Table 1:** Surface textural properties of raw GAC determined by BET analysis*.

Characteristic	Result
Single point surface area at p/p°	1200 m²/g
BET Surface Area	1106 m²/g
Langmuir Surface Area	1350 m²/g
t-Plot Micropore Area	701 m²/g
total pore volume	0.85 cm³/g
Adsorption average pore diameter	2.64 nm
Desorption average pore diameter	2.84 nm

*These values reflect a GAC with high surface area and pore volume, typical for high-quality activated carbon used in water treatment and adsorption applications.

### 4.1 Material properties and implications for reactivity

EDS confirmed Fe loading (~3 wt%), consistent with the target synthesis (Table 2), while FE-SEM revealed heterogeneous deposition of iron nanoparticles, increasing surface roughness and partially occluding micropore. Although partial pore blockage typically reduces surface area, the introduction of Fe–O functional groups (FT-IR) creates new reactive sites that can participate in complexation and redox reactions. This trade-off suggests that Fe–GAC performance is governed not solely by physical adsorption capacity but by coupled adsorption–catalysis mechanisms. The nano-scale iron domains are expected to facilitate electron shuttling and promote Fenton-like reactions, particularly under applied current. Thus, Fe–GAC should exhibit enhanced reactivity toward humic substances compared to pristine GAC, especially under non-equilibrium conditions.

**Table 2:** Elemental composition and distribution of iron nanoparticles immobilized on GAC as determined by EDS analysis.

Elt	Int	Error	K	Kr	W%	A%	ZAF	Pk/Bg	L. Conf^*^	H. Conf^*^
**C**	86.9	1.8626	0.3011	0.1108	41.33	53.30	0.2680	92.86	39.81	42.85
**O**	145.5	1.8626	0.2510	0.0923	36.98	35.81	0.2496	43.60	35.93	38.03
**Al**	225.7	2.1400	0.1365	0.0502	6.99	4.02	0.7177	21.44	6.84	7.15
**Si**	275.5	2.1400	0.1742	0.0641	8.44	4.66	0.7593	31.74	8.27	8.61
**S**	25.7	6.5472	0.0208	0.0077	0.95	0.46	0.8101	4.72	0.88	1.01
**K**	41.0	0.6635	0.0448	0.0165	1.96	0.78	0.8396	7.47	1.86	2.07
**Ca**	9.1	0.6635	0.0111	0.0041	0.48	0.18	0.8599	3.40	0.42	0.53
**Fe**	20.2	0.4818	0.0605	0.0222	**2.87**	0.80	0.7744	6.43	**2.65**	**3.09**
			1.0000	0.3678	100.00	100.00				

*Lower and upper confidence intervals (CI)

**Fig 3:**
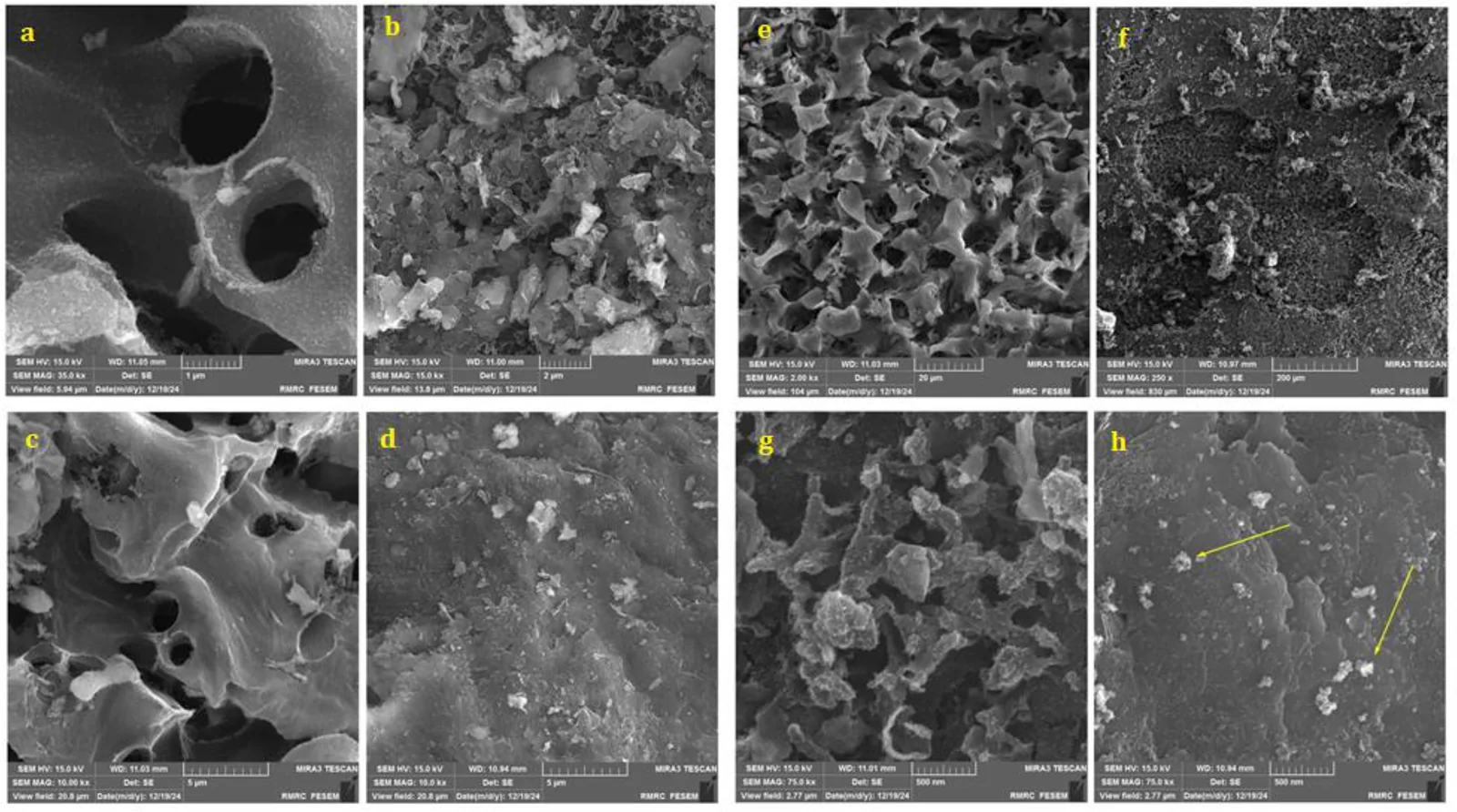
FE-SEM micrographs illustrating the surface morphology and structural characteristics of freshly synthesized Fe–GAC.

The size distribution of synthesized iron nano-particle-doped granular activated carbon showed that the particles range from approximately 300 nm at the smallest end to around 1,200 nm at the largest end, with the majority of particles concentrated sharply between about 600 nm and 900 nm. Almost all particles (≈100%) exhibited diameters below 1,200 nm, reflecting a relatively narrow and uniform size distribution within the submicron to low-micron range, which is favorable for efficient adsorption applications. A series of FE-SEM micro graphs (Fig 3a-3h) depicts iron nanoparticles loaded onto Fe-GAC at various magnifications. These nanoparticles, ranging from 50 to 100 nm in size, were observed to partially fill and coat the pores of the GAC, contributing to the roughened surface texture and the presence of particulate deposits. Lower magnification images (e.g., panels e, f, and h) provide a broader view of the Fe-GAC surface, showing more extensive nano-particle aggregation and highlighting the heterogeneity of iron nano-particle coverage. The arrows in panel h highlight distinct clusters of iron nanoparticles, underscoring their non-uniform distribution across the carbon surface. This morphology is characteristic of Fe-GAC composites synthesized via impregnation or co-precipitation methods, in which iron oxides or zero-valent iron nanoparticles (nZVI) adhere to the activated carbon surface but tend to agglomerate because of their high surface energy [23]. The observed partial pore blockage and surface roughening are consistent with the anticipated structural alterations resulting from iron nano-particle loading.

**Chart 3:**
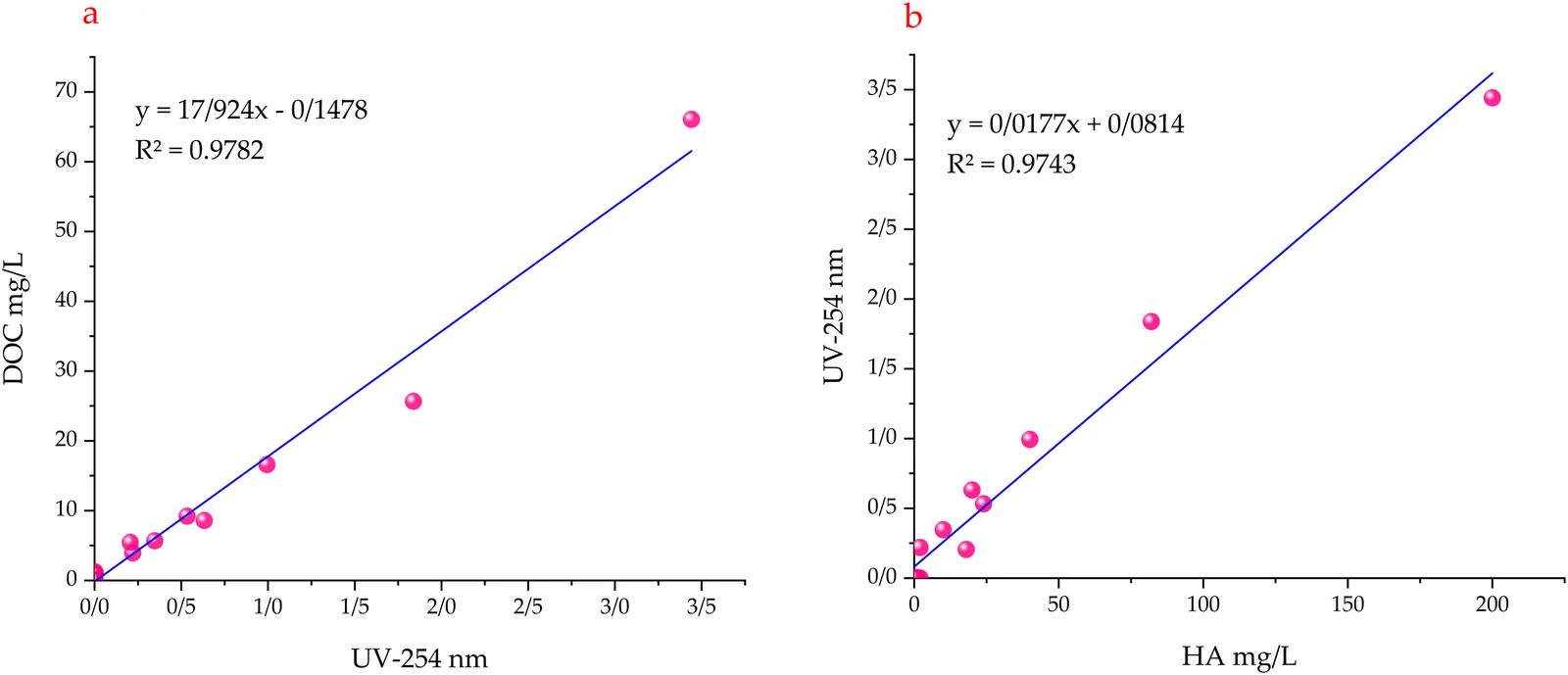
Relationships between adsorption kinetics and DOC removal efficiency (a), and correlation between humic acid concentration and DOC levels in treated samples (b).

Previous studies, such as that of Wang *et al.* [24] have reported that iron nanoparticles anchored on GAC surfaces form clusters that partially block micropore, leading to a decrease in BET surface area from pristine GAC to Fe-GAC (e.g., from ~941 to ~845 m²/g). This pore blockage is attributed to iron oxide nanoparticles occupying pore channels, which is visually supported by the SEM images showing pore coverage and particle agglomeration. Similarly, studies on Fe₃O₄@GAC composites used for electrochemicals degradation processes observed spherical iron oxide nanoparticles (~17 nm) uniformly distributed but also forming clusters on GAC surfaces, consistent with the granular deposits visible in this image. The magnetic properties of such composites, as confirmed by vibrating sample Magnetometry (VSM), are attributed to the presence of these iron oxide nanoparticles, which also influence surface morphology and pore accessibility. Iron nanoparticles appear as bright clusters on the carbon matrix, with partial pore filling and roughened surfaces [25,26]. These morphological modifications are associated with a decrease in pore volume and surface area, while simultaneously enhancing the adsorption and catalytic capabilities of the material for contaminants such as arsenic and organo-selenium compounds [27].

**Fig 4:**
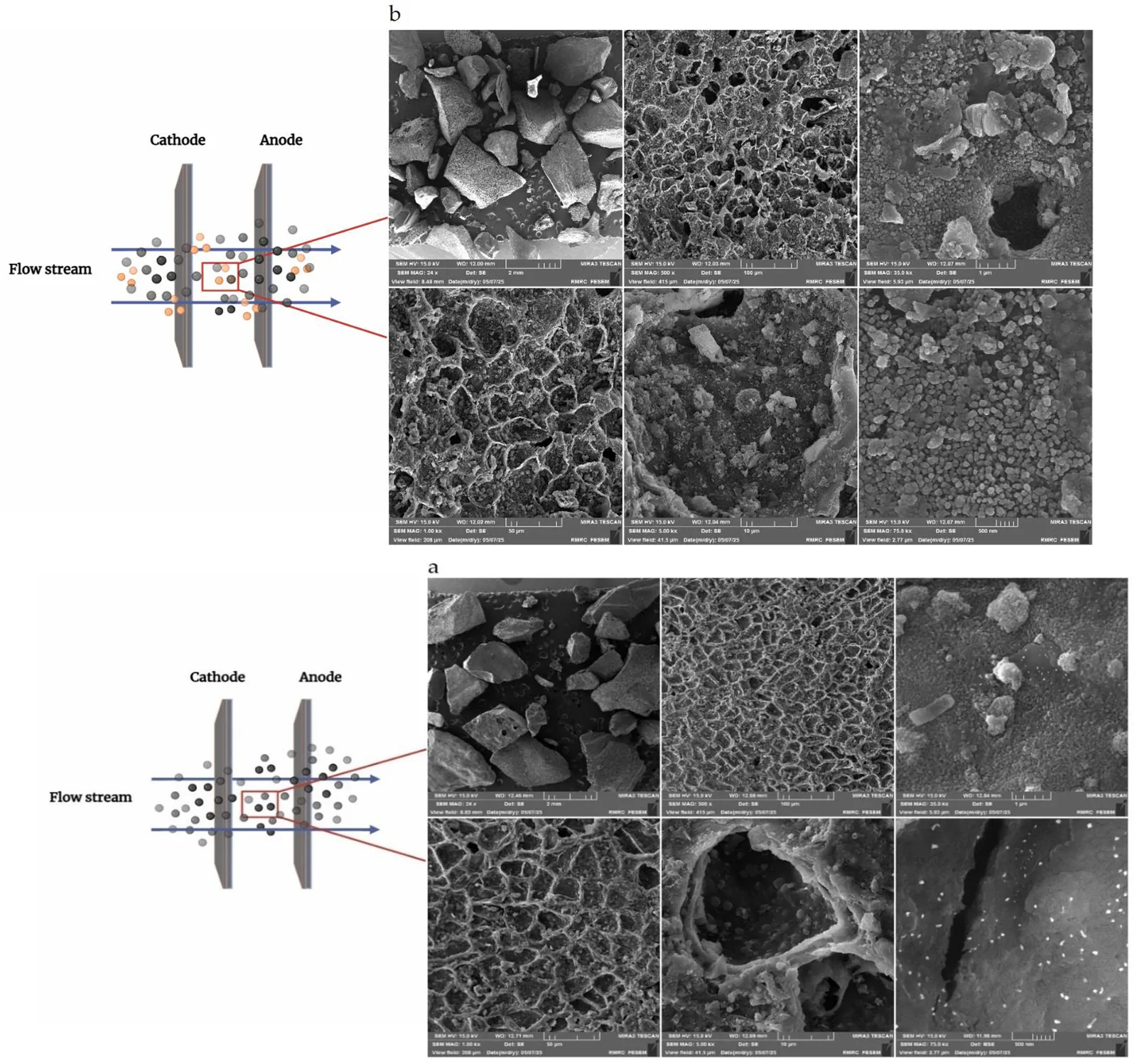
FE-SEM results of GAC (a) and Fe-GAC (b) after 4 months of operation for DOC removal. The sample was obtained from inside the column and between two electrodes (anode and cathode).

The six micrograph (Fig 4a), captured at various magnifications, reveal significant changes in the GAC’s surface morphology. The top-left image shows large, angular GAC fragments, offering some degree of particle attrition or breakage during prolonged use. The central and lower left images highlight a highly textured, web-like surface, indicative of substantial organic fouling, likely due to the accumulation of DOC and biofilm formation. The remaining panels display particulate deposits and evidence of microbial colonization, characterized by spherical and rod-shaped structures, further confirming the presence of biofilm and accumulated organic matter. The central bottom panel illustrates pore blockage, where organic residues and potential microbial byproducts occupy previously open channels. Fe-GAC micrograph generally exhibit roughened surfaces and partial pore blockage, along with distinctive features such as discrete clusters or coatings of iron nanoparticles, which occasionally form agglomerates that partially fill or line the pore structures. After months of DOC removal, Fe-GAC often exhibits both organic fouling and mineral deposits, as iron species can interact with organic matter to form precipitates or complexes. In contrast, the GAC image lacks the distinct particulate clusters typically associated with iron, and the fouling appears to be dominated by organic and biological materials rather than mineral or metal-organic complexes. Although both materials exhibit pore blockage and surface fouling, Fe-GAC tends to undergo more complex aging processes due to interactions between iron species and organic matter, which may result in distinct regeneration requirements and performance degradation patterns.

**Chart 4:**
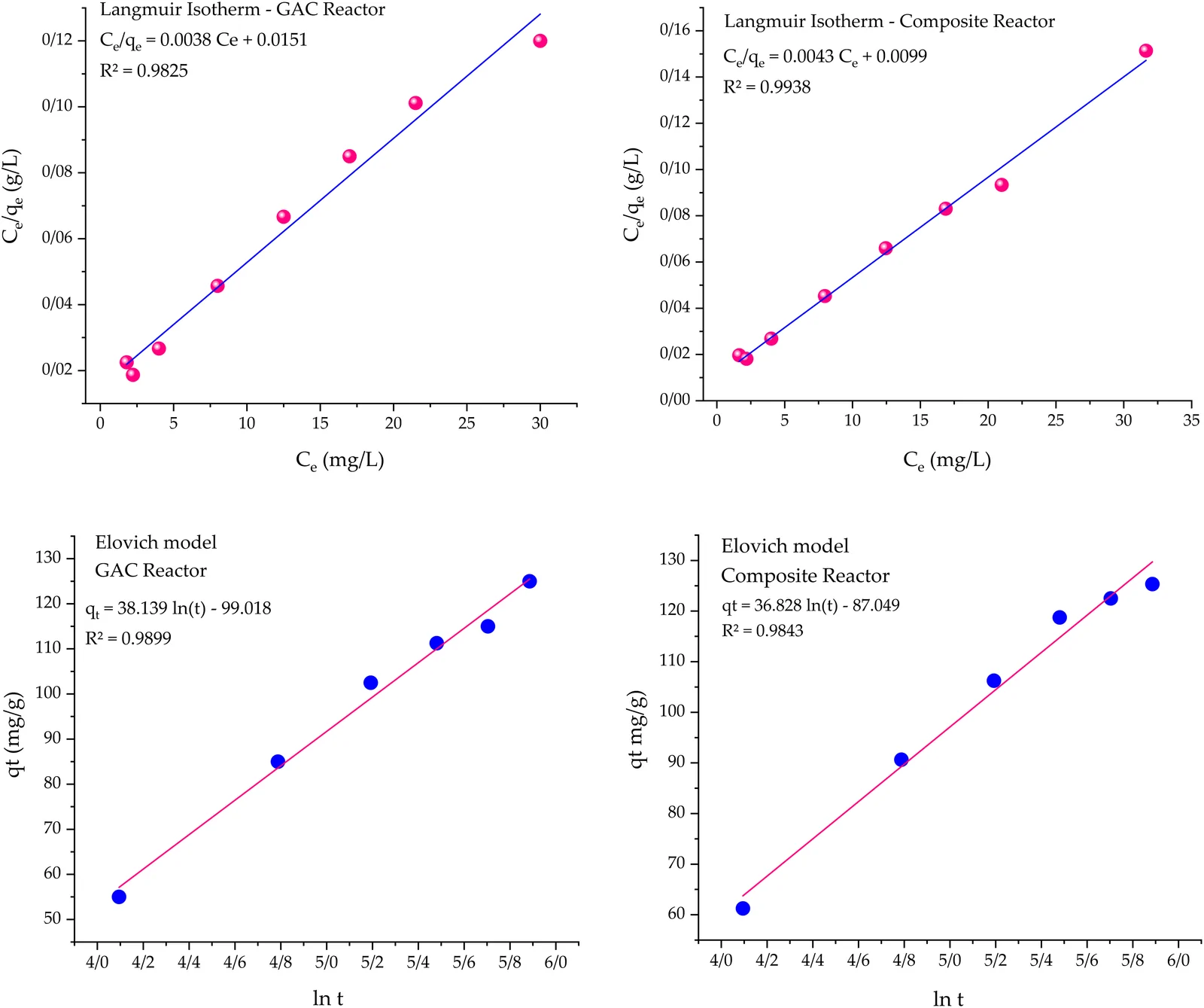
Adsorption isotherm and kinetic analysis of GAC and composite reactors.

The FE-SEM image (Fig 4b) of Fe-GAC after 4 months of operation for DOC removal from water display various magnifications and regions of the spent adsorbent. The top-left image shows fragmented, angular particles with surface morphology noticeably altered compared to the fresh Fe-GAC. The middle and right panels of both rows reveal pronounced surface alterations, including extensive biofilm formation, visible as web-like structures covering the carbon surface, and accumulated organic matter filling the pores. The bottom panels show how the original iron nanoparticles have been partially obscured or transformed, with spherical deposits and organic aggregates coating the previously distinct Fe particles. This fouling pattern is consistent with observations reported in other long-term studies on Fe-modified adsorbents. A study by Zhang *et al.* [28] demonstrated that after prolonged operation, Fe-GAC surfaces develop complex biofilm matrices that reduce adsorption capacity by blocking active sites and pores. The web-like structures visible in the middle panels closely resemble extracellular polymeric substances (EPS), which were examined for biological activity on iron-modified carbon materials [29,30]. The organic matter accumulation shown here has significant implications for the Fe-GAC performance lifespan. Similar to findings by Liu *et al.* [31], the extensive pore blockage indicates diminished DOC removal efficiency over time, as organic matter progressively occupies adsorption sites and reduces available surface area. The spherical deposits visible in the bottom-right panel indicate possible precipitation of iron complexes with organic matter, a phenomenon also reported by Wang *et al.* [32] for humic acid removal with Fe-GAC composite. This observation effectively illustrates the complex aging behavior of Fe-GAC in water treatment applications, showing how the initially distinct iron nanoparticles become incorporated into a heterogeneous matrix of organic deposits, precipitates, and biofilm, ultimately influencing the adsorbent’s regeneration requirements and operational lifespan [33]. Using electrodes with a high specific surface area in flow-through mode, where water flows directly through the electrode pores, greatly enhances the reaction rates. The increase is due to water being convectively transported through the electrode’s tiny pores, which reduces the thickness of the diffusional boundary layer to a size similar to the pore radius. Additionally, the surface roughness of the electrode occurs on a scale comparable to the diffusion field, rendering a substantial portion of the surface electro-active. Collectively, these factors result in reaction rate constants for microporous electrodes that are an order of magnitude higher in flow-through mode compared to flow-by or batch configurations [34,35].

The combination of GAC with iron not only improves adsorption capacity but also introduces magnetic properties that facilitate easier recovery from solutions. FT-IR spectroscopy plays a vital role in characterizing these materials and optimizing their performance in environmental applications. The results of this test about Fe-GAC are shown in Chart 2, based on the wavenumber (cm^-1^) and transmittance percent (%). The peaks near 588 cm ⁻ ¹ were attributed to Fe–O vibrations in magnetite (Fe₃O₄), whereas other iron oxides such as goethite (FeO(OH)) exhibited characteristic peaks at 615 cm ⁻ ¹ and 798 cm ⁻ ¹. A broad peak centered at 1424 cm ⁻ ¹ was observed in Fe–GAC and is attributed to O–H bending vibrations arising from carboxylic acid or alcohol groups. Additionally, the broad band around 1080 cm ⁻ ¹ can be assigned to C–H and C = C bending vibrations. The band observed at 665 cm ⁻ ¹ confirms the successful doping of Fe₂O₃ nanoparticles onto the GAC surface, corresponding to the presence of FeOOH functional groups. The appearance of similar absorption peaks in both raw GAC and Fe–GAC indicates that the GAC structure was preserved after modification, as evidenced by the formation of new functional groups on the surface. Moreover, similar spectral patterns have been reported in the literature, suggesting that the modification process disrupts organic structures and increases the abundance of acidic functional groups on the GAC surface [30]. Therefore, more acidic groups enhance the number of active binding sites, which might offer more adsorption sites for binding Pb(II) and Cr(T) ions.

The concentration details of various soil elements detected in parts per billion (ppb) through digestion and ICP-OES analysis, comparing blank controls with a natural soil sample used as a microbial source. The results (**See** S1 Table in S1 File) indicate that key macro-elements, including aluminum (Al), calcium (Ca), iron (Fe), magnesium (Mg), and potassium (K), are present in extremely high concentrations in the soil sample, significantly exceeding blank values by orders of magnitude. Trace elements and potential micronutrients, such as arsenic (As), copper (Cu), nickel (Ni), lead (Pb), and zinc (Zn), also demonstrate marked elevation in the soil samples relative to blanks. Arsenic, for instance, rises from around 1.3 ppb in blanks to 130 ppb in soil, while copper is nearly 23 times higher in the sample (459.9 ppb versus 20.1 ppb).

The relationships between the adsorption rate and the concentration of DOC (Chart 3a), the adsorption rate and the concentration of humic acid (Chart 3b) in the sample showed that about 97.82% of the DOC removal rate using adsorbents could be predicted using the absorption rate equation (y = 17.924x −0.1478) at a wavelength of 254 nm. Also, 97.43% of the concentration of humic acid based on the absorption rate equation (y = 0.0177x + 0.0814) was predictable at λ = 254 nm.

The adsorption equilibrium data for both the GAC and composite reactors were analyzed using the Langmuir isotherm model, and the corresponding linearize plots are presented in Chart 4. The Langmuir model showed excellent agreement with the experimental data for both adsorbents, with correlation coefficients (R²) of 0.9825 and 0.9938 for the GAC and composite reactors, respectively. The higher R² value obtained for the composite reactor indicates a better fit to the Langmuir model, suggesting that adsorption occurred predominantly through monolayer coverage on a homogeneous surface. The Langmuir constants further revealed differences in adsorption characteristics between the two adsorbents. The lower intercept observed for the composite reactor suggests a stronger adsorbate–adsorbent affinity compared to GAC, indicating the presence of more favorable adsorption sites within the composite material. Although the GAC reactor exhibited a slightly lower slope, implying a marginally higher theoretical maximum adsorption capacity, the composite reactor demonstrated superior adsorption affinity and a more uniform adsorption behavior.

**Chart 5:**
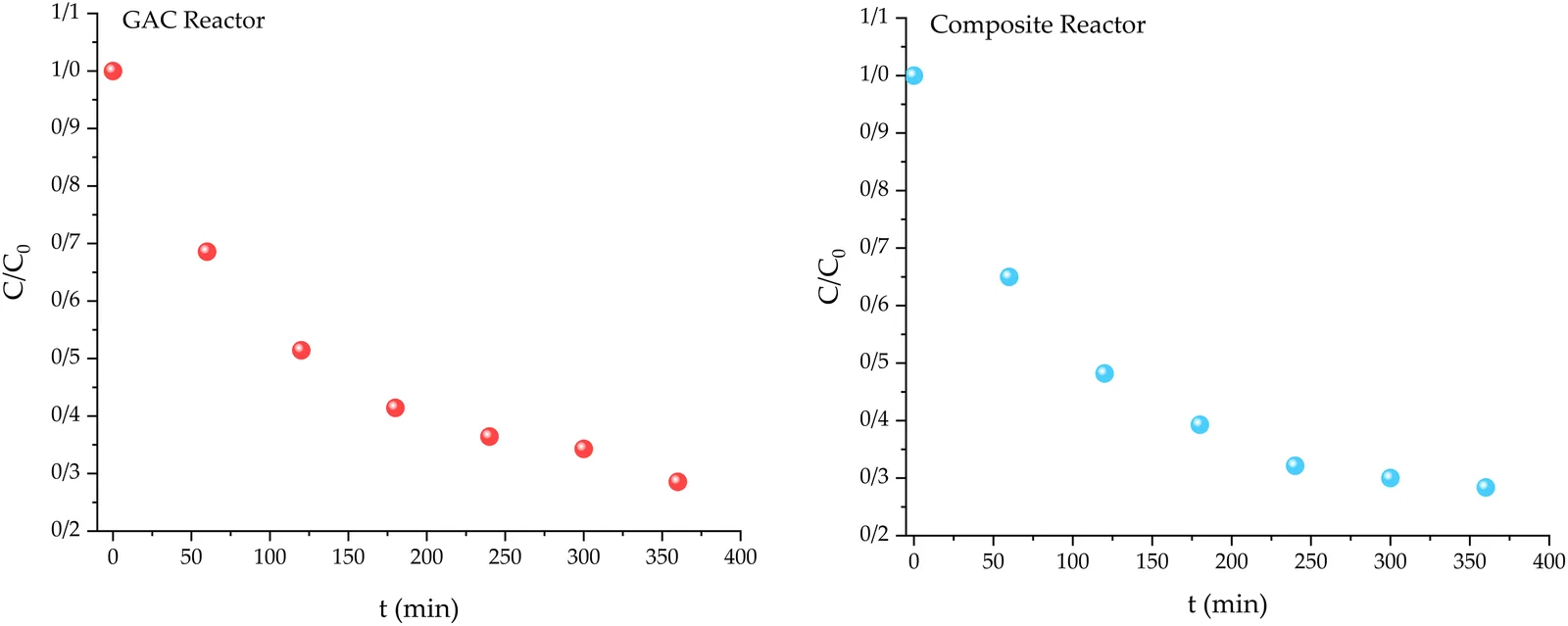
Elovich kinetic model and concentration variations of GAC and Composite Reactors.

The adsorption kinetics were evaluated using the pseudo-second-order (PSO) kinetic model, as shown in the same Chart 5. The linear relationships between t/qt and contact time yielded high correlation coefficients of 0.9963 and 0.9976 for the GAC and composite reactors, respectively. These results indicate that the PSO model adequately describes the adsorption process for both systems. The slightly higher R² value obtained for the composite reactor suggests that its adsorption mechanism is more accurately represented by the PSO model. Furthermore, the lower intercept value observed for the composite reactor corresponds to a higher calculated equilibrium adsorption capacity (qe), indicating enhanced adsorption performance compared to GAC. The excellent agreement between experimental and modeled data also suggests that surface interactions play a significant role in controlling the adsorption rate.

The findings obtained in this study are consistent with previous reports on activated carbon and composite adsorbents [36,37], where the Langmuir isotherm and PSO kinetic models frequently provide the best description of adsorption behavior. GAC exhibits effective adsorption due to its high surface area and porous structure, while composite materials often demonstrate improved adsorption performance through the incorporation of additional functional groups and active sites. The superior Langmuir and kinetic fitting observed for the composite reactor in the present study indicates that the modification of GAC into a composite material enhanced the uniformity of adsorption sites and strengthened adsorbate–surface interactions. Consequently, the composite reactor exhibited more favorable adsorption characteristics and kinetic performance than the conventional GAC reactor, highlighting its potential as an efficient adsorbent for water treatment applications.

The Elovich kinetic model adequately described the adsorption behavior of both GAC and composite reactors, with high correlation coefficients (R² = 0.9899 for GAC and 0.9843 for the composite). These results indicate that adsorption occurred on heterogeneous surfaces and was governed predominantly by chemisorption mechanisms. The slightly higher R² value for GAC suggests a stronger influence of surface heterogeneity, whereas the composite adsorbent may possess a more uniform distribution of active sites resulting from the modification process.

The adsorption capacity (qt) increased steadily with contact time for both adsorbents, while the normalized concentration (C/C₀) decreased continuously, confirming effective contaminant removal. Although both systems exhibited comparable final removal efficiencies after 360 min, the composite reactor achieved lower C/C₀ values during the intermediate stages of adsorption, indicating faster adsorption kinetics and enhanced mass-transfer characteristics. This improvement can be attributed to the additional functional groups and active adsorption sites introduced into the composite structure. Compared with previous studies, the obtained Elovich correlation coefficients are within the range commonly reported for activated carbon and carbon-based composite adsorbents (R² > 0.95), confirming the applicability of the model for heterogeneous adsorption systems. However, unlike several studies that reported substantially higher adsorption capacities and removal efficiencies for modified composite adsorbents, the improvement observed in the present study was relatively moderate. While the composite reactor exhibited faster adsorption rates and slightly better overall performance than GAC, the final contaminant removal efficiencies of both reactors were very similar. This finding suggests that the modification of GAC improved adsorption kinetics more effectively than adsorption capacity. The discrepancy between the present results and some published studies may be attributed to differences in adsorbent composition, surface chemistry, pore structure, operating conditions, contaminant type, and initial concentration [38,39]. Many studies reporting significant improvements in composite adsorbents employed nano-materials, metal oxides, or highly functionalized surfaces that greatly increased the number of available active sites. In contrast, the composite used in this study appears to enhance adsorption efficiency without dramatically altering the ultimate adsorption capacity. Therefore, the results demonstrate that while the composite reactor offers kinetic advantages over conventional GAC, further optimization of the composite formulation may be required to achieve the substantial performance enhancements reported in other adsorption studies [40].

**Chart 6:**
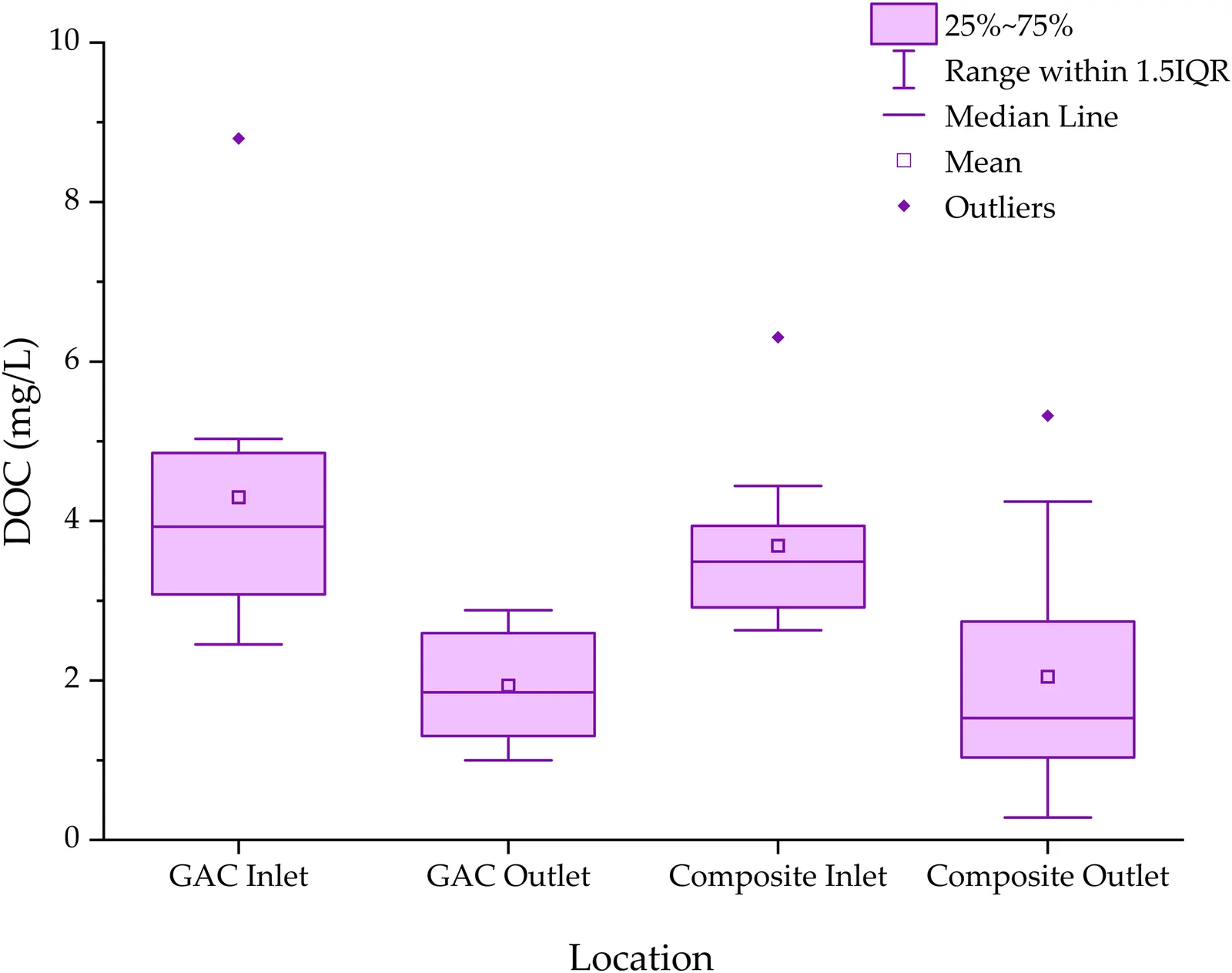
Box plot of DOC concentration (mg/L) for the GAC and Fe-GAC reactors for 40 samples (a: without outliers, and b: with outliers).

Chart 6 compares DOC concentrations at the inlet and outlet of both reactors for 40 pairs of samples in three phases. In all cases for the GAC reactor, inlet DOC concentrations are consistently higher than outlet values, demonstrating effective DOC removal by the GAC column. The data show variability in inlet DOC concentrations, ranging from approximately 1–18 mg/L, while outlet concentrations are generally lower, ranging from about 0–6 mg/L. The consistent reduction in DOC from inlet to outlet underscores the effectiveness of the GAC column in removing dissolved organic carbon from water. Similarly, in the Fe-GAC reactor, outlet DOC concentrations were consistently lower than inlet values across most samples, confirming the strong DOC removal capability of the Fe-GAC column. There were some fluctuations in the inlet concentrations, with a few samples showing notably higher DOC levels, but the outlet values remained relatively low and consistent in comparison. This trend further demonstrates the Fe-GAC column’s high efficiency in reducing DOC concentrations across the tested range of water samples. Also, DO concentration was checked alternatively for each phase and the results (See **Table S2** in S1 File) showed the composite reactor had less oxygen concentration in contrast of GAC reactor.

The pH variations of influent and effluent samples treated with GAC and composite (See Chart S1 in S1 File) showed variable effects on pH, whereas Fe-GAC produced more stable effluent pH values and demonstrated better buffering capacity. The lower variability observed in the Fe-GAC effluent suggests improved pH regulation despite fluctuations in influent water quality. Similar findings have been reported by Lai *et al.* [41], highlighting the importance of pH control in enhancing treatment performance. Detailed pH data and trends are provided in the Supporting Information.

Descriptive statistics for water quality parameters before (inlet) and after (outlet) treatment in reactors, based on analysis of physicochemical parameters of 25 samples presented in Table 3. DOC decreased significantly from the inlet (mean = 4.26 ± 3.21 mg/L) to the outlet (mean = 2.56 ± 1.08 mg/L), demonstrating the GAC’s effectiveness in organic removal. The pH showed a slightly increased from 7.68 to 7.78 but exhibited greater variability (SD = 0.45–0.65). TDS demonstrated a modest reduction from 259.84 to 248.46 mg/L, accompanied by increased variability at the outlet (SD 40.30 to 65.37). In contrast, EC exhibited pronounced outlet fluctuations with mean values rising from 0.51 to 10.69 µS/cm, largely influenced by an extreme maximum reading of 255 µS/cm. The data highlights the strong DOC removal capacity of GAC, accompanied by variable effects on other water quality parameters. Also, the second section shows descriptive statistics for water quality parameters in the Fe-GAC reactor for about 25 samples, comparing inlet and outlet measurements. DOC decreased substantially from the inlet (mean = 3.61 ± 1.68 mg/L) to the outlet (mean = 1.64 ± 1.34 mg/L), demonstrating Fe-GAC’s enhanced organic removal compared to standard GAC. When the pH increased in standard GAC, Fe-GAC demonstrated a reduction in pH (7.72 to 7.14) while stabilizing variability (SD = 0.46–0.27). The results regarding TDS showed a clearer reduction (263.32 to 241.08 mg/L) with decreased outlet variability (SD = 49.90 to 43.91), and EC remained stable (0.52 to 0.47 µS/cm), without extreme outliers observed in the standard GAC. Overall, Fe-GAC improved DOC removal efficiency and parameter stability, especially supporting bacterial growth, compared to conventional GAC, particularly in pH regulation and conductivity control, and it was significantly more effective than adsorption by DOC alone across all tested conditions [42].

**Table 3:** Comparative descriptive statistics of water quality parameters measured at the influent and effluent of the reactors.

Parameter (GAC reactor)	N	Min.	Max.	Mean ± SD
DOC GAC Inlet (mg/L)	25	1.19	17.20	4.25 ± 3.21
pH Inlet	25	6.80	8.38	7.67 ± 0.45
TDS Inlet (mg/L)	25	229	428	259.84 ± 40.30
EC Inlet (µS/cm)	25	0.44	0.85	0.50 ± 0.08
DOC GAC Outlet (mg/L)	25	0.00	4.87	2.55 ± 1.07
pH Outlet	25	6.80	8.91	7.77 ± 0.65
TDS Outlet (mg/L)	25	0.50	417.00	248.46 ± 65.37
EC Outlet (µS/cm)	25	0.38	0.86	0.55 ± 0.13
**Parameter (Fe-GAC reactor)**	**N**	**Min.**	**Max.**	**Mean ± SD**
DOC GAC Inlet (mg/L)	25	1.16	10.24	3.61 ± 1.68
pH Inlet	25	6.80	8.42	7.72 ± 0.45
TDS Inlet (mg/L)	25	230	432	263.32 ± 49.90
EC Inlet (µS/cm)	25	0.45	0.850	0.52 ± 0.09
DOC GAC Outlet (mg/L)	25	0.00	6.28	1.63 ± 1.33
pH Outlet	25	6.40	7.54	7.14 ± 0.27
TDS Outlet (mg/L)	25	184	405	241.08 ± 43.91
EC Outlet (µS/cm)	25	0.36	0.80	0.47 ± 0.08

*N is the sample volume, and SD is the standard deviation

Statistical analysis (Table 4) demonstrated significant differences between influent and effluent water quality parameters in both treatment configurations. For the GAC system, DOC concentrations were significantly reduced after treatment, as confirmed by the Wilcoxon signed-rank test (W = 21.0, p < 0.001), with a very large effect size (r = 0.817), indicating a substantial treatment impact on organic carbon removal. Likewise, a statistically significant change in pH was observed between the inlet and outlet streams (paired t-test, t = 2.34, p = 0.0246), although the effect size was moderate (Cohen’s dz = 0.365), suggesting a less pronounced influence on pH compared with DOC removal. In the composite treatment system, DOC removal was also highly significant (W = 45.0, p < 0.001), with a large effect size (r = 0.752), confirming the strong capability of the integrated process to reduce dissolved organic matter. Furthermore, pH exhibited a highly significant change between influent and effluent samples (W = 9.5, p < 0.001), accompanied by a very large effect size (r = 0.830), indicating a substantial alteration in water chemistry during treatment. Based on the statistical results both treatment systems effectively modified water quality parameters, with particularly strong and consistent effects observed for DOC removal, while the composite system exerted a more pronounced influence on pH than the standalone GAC process.

**Table 4:** Results of the wilcoxon signed-rank test and paired t-test analyses.

Comparison	Appropriate Test	Test Statistic	*p*	Effect Size	Interpretation
DOC GAC Inlet vs Outlet	Wilcoxon Signed-Rank Test	W = 21.0	< 0.001	r = 0.817	Very large effect
pH GAC Inlet vs Outlet	Paired t-test	t = 2.34	0.0246	Cohen’s dz = 0.365	Moderate effect
DOC Composite Inlet vs Outlet	Wilcoxon Signed-Rank Test	W = 45.0	< 0.001	r = 0.752	Large effect
pH Composite Inlet vs Outlet	Wilcoxon Signed-Rank Test	W = 9.5	< 0.001	r = 0.830	Very large effect

**Chart 7:**
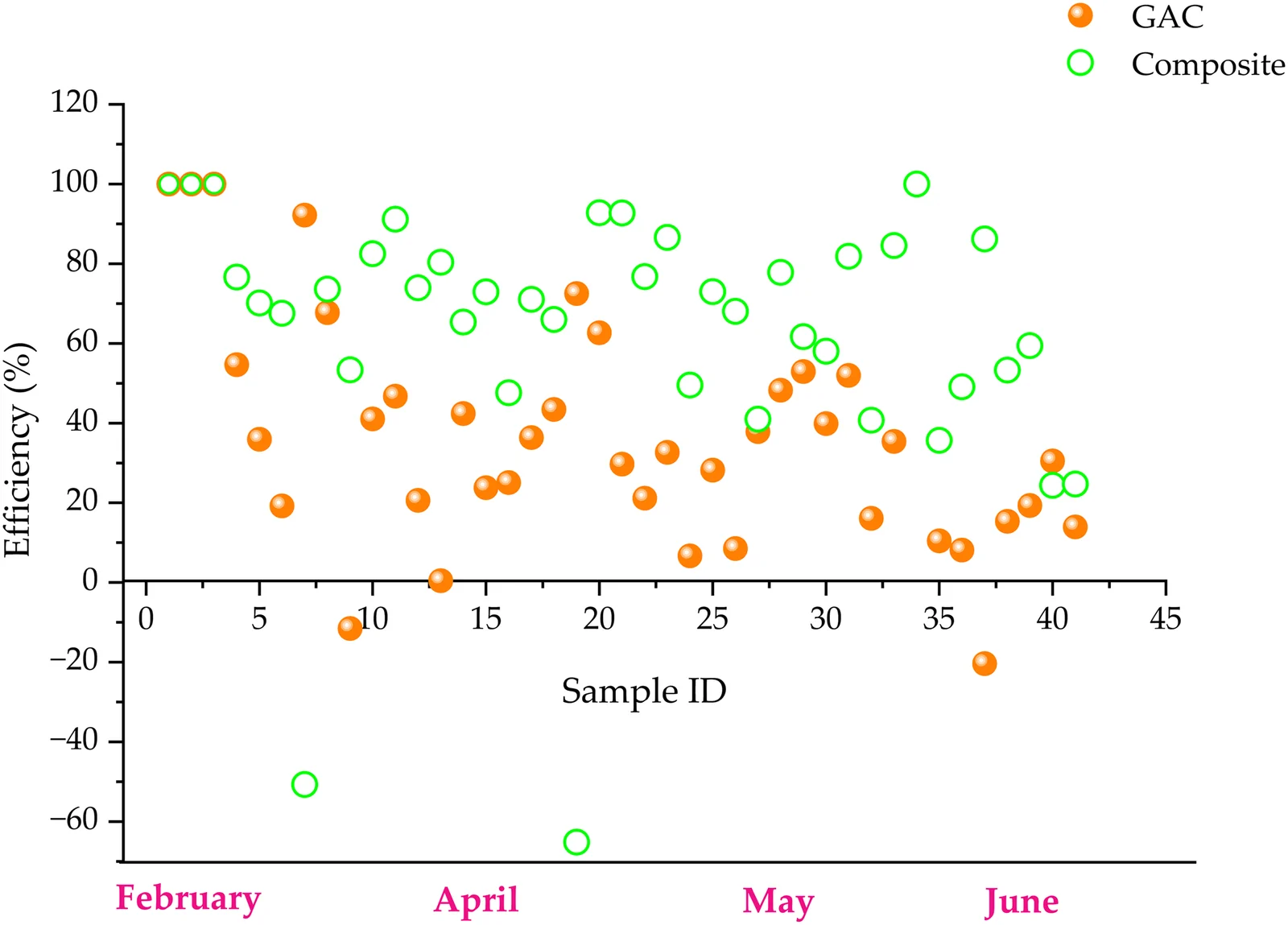
DOC removal performance of reactors during the physicochemical, biological, and bioelectrochemical operational phases.

Based on the efficiency results (Chart 7) GAC reactor exhibited a notable reduction, with efficiencies dropping to a range between 0% and 60%, and some values even turned negative, indicating occasional DOC release rather than removal. The Fe-GAC Reactor, while showing a decrease, generally maintains higher efficiencies than the GAC Reactor, with most values between 20% and 80%. This proves that Fe-GAC has a more sustained removal capability, due to additional adsorption or catalytic mechanisms provided by the iron component. In the bio-electrochemical phase (phase III), the declining efficiency trend persisted, yet the performance gap between the two reactors became more pronounced. After saturation and biofilm development, GAC may release previously adsorbed DOC back into the water (desorption), leading to negative removal efficiencies and deterioration of effluent water quality [42–44]. In contrast, the Fe-GAC reactor, although exhibiting a few negative values, generally maintains superior performance, with most points above 0% and several still achieving moderate removal (20–60%). This phase highlights the enhanced longevity and stability of the Fe-GAC system under extended operation. Although individual components such as biofilm formation on GAC or GAC embedded with iron for Fenton-type reactions have been previously investigated, the sequential integration of all three processes within column reactors represents a novel concept. The results demonstrate that GAC with iron outperforms plain GAC, particularly in later stages when adsorption sites are saturated, highlighting the important role of iron nanoparticles coupled with GAC [45,46]. Therefore, it can be confirmed that adding iron nanoparticles did not negatively affect the adsorption capacity of the GAC, and this synergistic approach not only maximizes DOC removal but also reduces the formation of DBPs [47].

**Chart 8:**
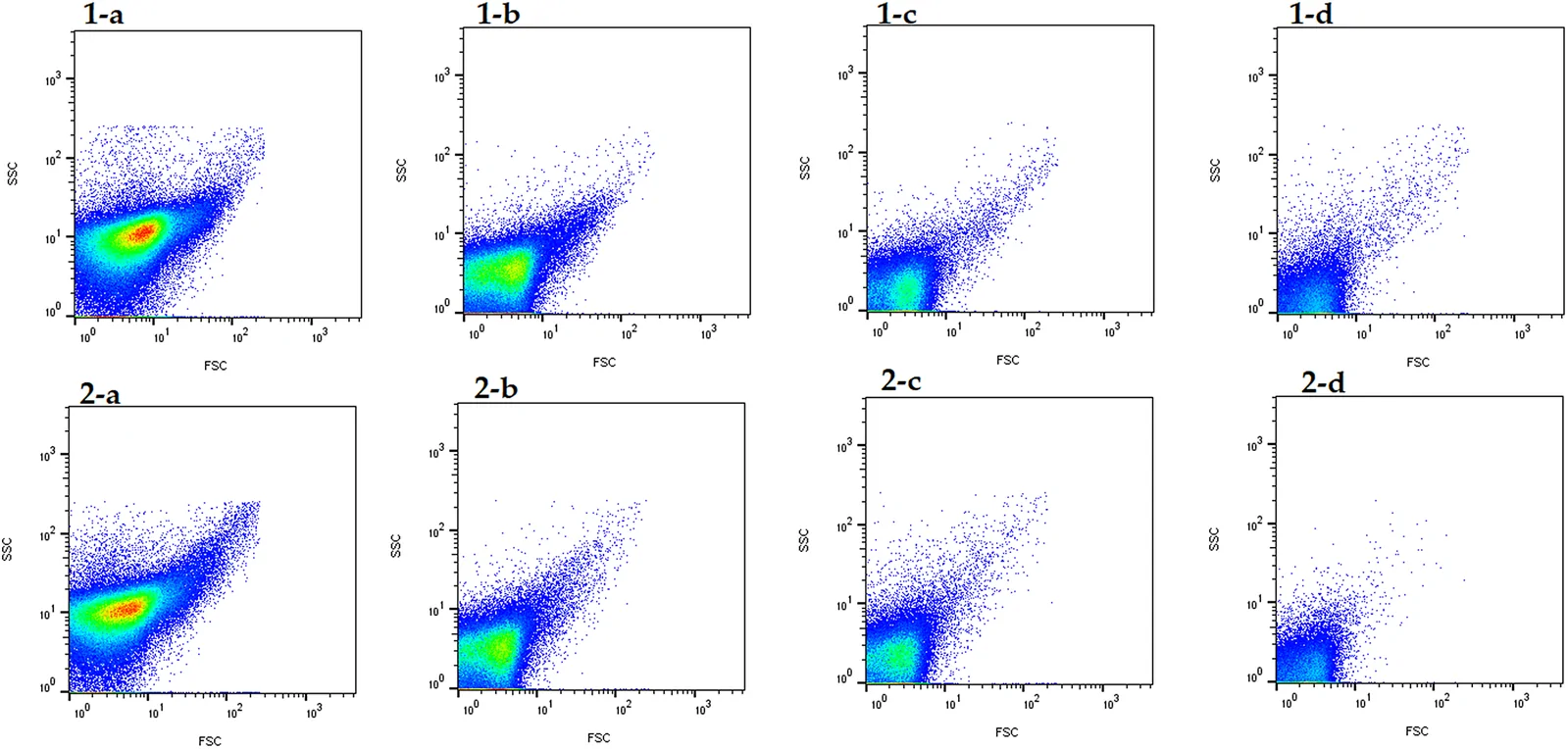
The flow cytometry dot plot of water samples from the columns, which were stained with acridine orange—samples 1-a to 1-d from the Fe-GAC column, with in serial dilution order, and samples 2-a to 2-d from the GAC column in serial dilution order.

Previously, total cell enumeration primarily relied on microscopic methodologies. However, recent advancements in epi-fluorescence microscopy, the development of diverse fluorophores, and sophisticated digital imaging techniques now provide highly accurate and efficient alternatives. Consequently, this study employed FCM to achieve rapid and precise quantification of bacterial cell populations [48]. The flow cytometry dot plots (Chart 8) compare water samples filtered through two columns: Fe-GAC (1-a to 1-d) and GAC (2-a to 2-d), each presented in a serial dilution series. In both datasets, the undiluted samples (1-a and 2-a) show a high density of events, indicated by the intense red and green regions in the lower left quadrant of the plots, which correspond to populations with lower forward scatter (FSC) and side scatter (SSC)—typically representing smaller or less complex particles, such as bacteria stained with AO. The stain effectively highlights bacterial cells by fluorescence, enabling accurate manual counting under epifluorescence microscopy [49,50]. As the dilution increases (from -a to -d), both Fe-GAC and GAC samples show a reduction in event density, as expected. However, the Fe-GAC series (1-a to 1-d) appears to have a slightly lower overall event density compared to the GAC series (2-a to 2-d) at corresponding dilution levels. This findings suggests that the Fe-GAC column may be more effective in removing or reducing the concentration of particles or microorganisms in the water. The difference is most notable in the more concentrated samples (1-a vs. 2-a and 1-b vs. 2-b), where the Fe-GAC plots show a less intense signal, indicating fewer detected events. This observation aligns with the known adsorptive and potentially antimicrobial properties of iron nanoparticles, which can enhance the removal efficiency of activated carbon filters. The water quality significantly influences bacterial dynamics and treatment efficacy in drinking water systems, with flow cytometric total bacterial cell counts revealing microbial changes not detected by traditional monitoring methods [48].

Microscopic images of biofilms in the GAC and Fe-GAC reactors are shown in S1 Fig in S1 File. Gram stain and acridine orange staining confirmed dense bacterial growth on both media. Detailed microscopic observations are provided in the Supporting Information.

AO staining provides a more comprehensive visualization of total biomass, including cells embedded in the matrix and extracellular DNA, resulting in a more diffuse and widespread fluorescence signal [51,52]. On the other hand, iron nanoparticles often promote even greater bacterial aggregation and activity due to the presence of iron, which can stimulate microbial growth and electron transfer processes [53,54]. The enhanced visualization of biofilms with AO is particularly valuable in reactor studies, as it allows for the assessment of both viable and non-viable cells, as well as the structural integrity of the biofilm. This aspect is crucial for assessing reactor performance, as biofilm stability and density are strongly correlated with overall treatment efficiency [55]. Furthermore, the beneficial effects of iron nanoparticles—highlighted by the denser clusters in Fe-GAC samples—are substantiated by recent studies [56–58] that iron fosters microbial activity by acting as an electron donor in redox processes and supporting the establishment of diverse microbial colonies, especially under anoxic or oligotrophic conditions. Iron-enhanced biofilms often display superior pollutant removal rates and resilience against environmental fluctuations, outcomes verified by both microscopy and performance metrics in peer-reviewed reactor trials. Also, Iron nanoparticles exhibit properties that help regulate microbial populations and biofilm growth, preventing excessive fouling. When combined with electrochemical stimulation, Fe-GAC systems maintain enhanced DOC removal efficiency by limiting biofilm-related performance decline [59,60].

**Fig 5:**
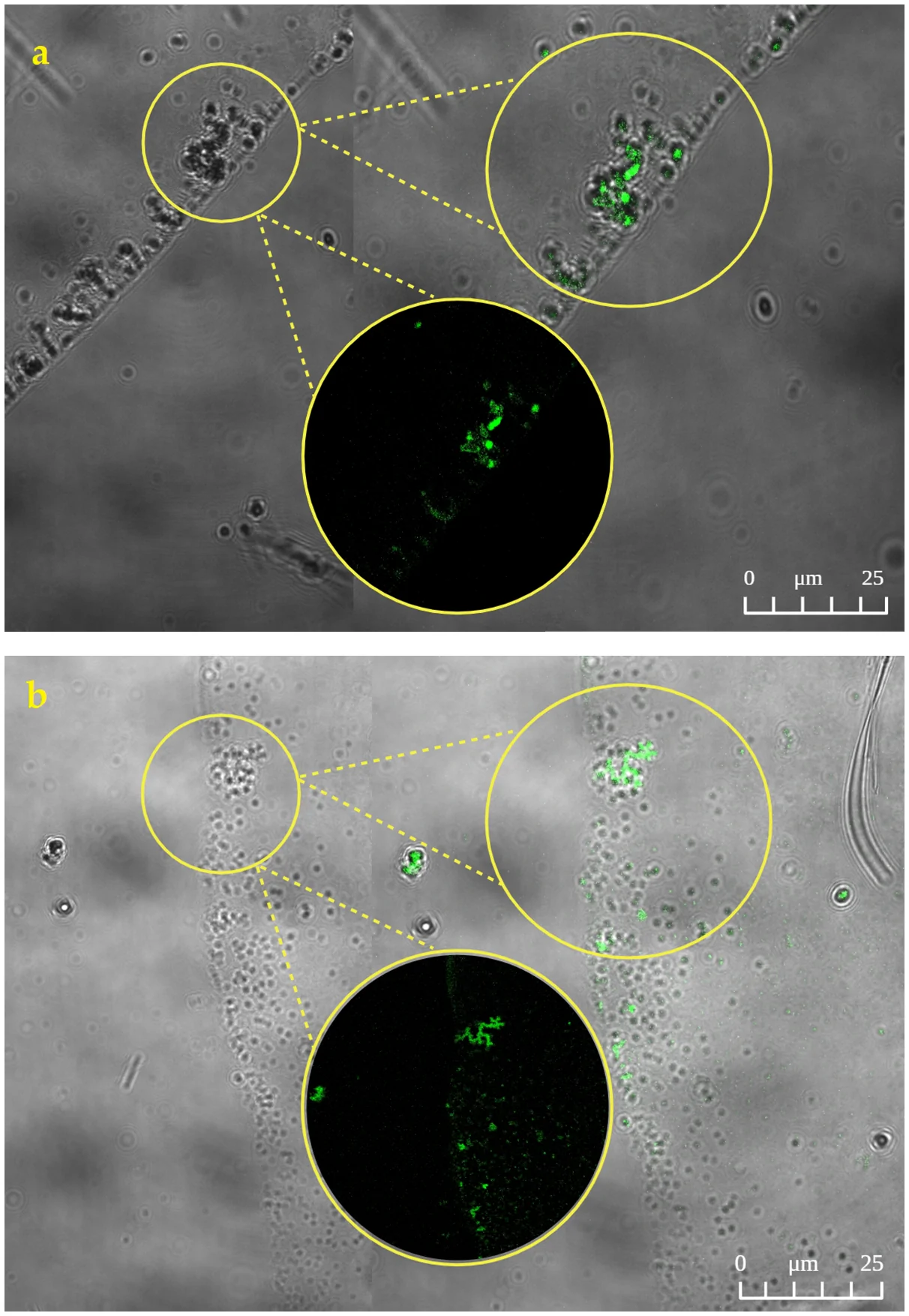
The CLSM images for GAC (a) and Fe-GAC (b) samples, which were stained with acridine orange, 25 µm in 25 µm dimensions.

In a GAC filter, DOC removal primarily occurs through two mechanisms: adsorption onto the GAC surface and biodegradation mediated by the microorganisms colonizing it [61]. In this study, Fig 5 presents CLSM images comparing GAC (a) and Fe-GAC (b) samples, both stained with AO. The GAC sample (a) shows localized clusters of fluorescence, while the Fe-GAC sample (b) displays a different distribution, potentially reflecting the influence of iron modification on surface properties or microbial colonization. These visual differences underscore the impact of iron nanoparticle treatment on the microenvironment of GAC, as revealed by the spatial distribution of AO-stained components. The use of CLSM combined with acridine orange is invaluable for distinguishing live, dead, and extracellular DNA components within biofilms, providing detailed spatial information that conventional microscopy cannot achieve. The denser and more aggregated green fluorescence observed in Fe-GAC samples corroborates findings that iron amendments in carrier materials significantly enhance microbial colonization, spatial organization, and biofilm thickness—attributes that underpin improved pollutant removal and reactor efficiency [62]. Furthermore, the images reveal how Fe-GAC supports multilayer bacterial communities and extracellular matrix formation, contributing to biofilm resilience and metabolic diversity in engineered systems, trends widely validated in advanced water treatment literature.

These findings indicate that iron impregnation not only alters the physicochemical properties of the carbon surface but also affects the distribution of microbial or organic matter, thereby enhancing the material’s performance in environmental applications such as water treatment or bioremediation [63]. In the previous studies [48]FCM measurement was conducted on the liquid phase of samples, whereas in the present study, cell counts were attributed to the biomass attached to the GAC. In addition to FCM, the centrifuged samples were distributed throughout the reactors and observed with the CLCM for biomass confirmation of biomass formation on the composites. These results offer new insights by quantifying the effects of iron saturation and mild electrochemical stimulation on long-term DOC removal and biofilm development. It demonstrates that Fe-GAC not only improves initial adsorption but also provides catalytic or electrochemical advantages that help reduce performance loss caused by biofouling. Using CLSM images after four months of operation, along with flow cytometry and cell counting, the study sheds light on the interactions between biofilms and iron nanoparticles during extended use, an area that has been little explored in DOC treatment research. The data also suggest that the presence of iron may have some antimicrobial or growth-regulating effects, as the GAC-Fe column showed a slightly lower number of microbial cells in the effluent.

Several mechanistic aspects remain beyond the scope of the present study. Although Fe-GAC demonstrated improved DOC removal and greater operational stability than conventional GAC, direct evidence for catalytic oxidation, iron redox cycling, extracellular electron transfer, and microbial metabolic activity was not obtained. Advanced characterization techniques such as XPS, cyclic voltammetry, electrochemical impedance spectroscopy, metagenomic sequencing, and EPS quantification would provide valuable mechanistic insights and should be pursued in future investigations.

## Conclusion

This study demonstrates that Fe–GAC significantly enhances DOC removal performance and stability in continuous-flow systems. Unlike conventional GAC, Fe–GAC maintains efficiency across physicochemical, biological, and bioelectrochemical phases. Fe-GAC consistently outperformed GAC in terms of both average efficiency and operational stability, especially as the number of samples increased and the systems aged. The negative efficiencies observed in later phases, particularly for GAC, indicate not just exhaustion of adsorption sites but also potential desorption or release of previously retained DOC, which is a critical operational concern. The observed performance may be associated with additional adsorption sites, redox-active iron species, and possible electrochemical interactions; however, these mechanisms were not directly measured in the present study. These results underscore the importance of material modification (such as iron impregnation) for improving the long-term efficacy and reliability of adsorptive water treatment systems, particularly for persistent organic contaminants like DOC. The improved performance is attributed to synergistic interactions between adsorption, catalytic processes, and bioelectrochemical activity. Additionally, Fe–GAC regulates biofilm growth, reducing fouling and preventing performance deterioration.

## Supporting information

S1 File Supporting information containing Table S1, Table S2, Chart S1, and Figure S1. (DOCX)
