# Supplementary material for: A multi-phase reactions for organic carbon removal in bio-electrochemical reactors employing Fe2O3-synthesized nanoparticles
Source: PLoS One. 2026 Aug 20;21(8):e0355332. doi: 10.1371/journal.pone.0355332 (PMC13492793; doi:10.1371/journal.pone.0355332)
Supplement: Supplementary file 1 [file pone.0355332.s001.docx]

**Table S1** demonstrates the elemental content of 1mg of soil used as a microbial source in the nutritional supplement for feeding the reactors for a short duration in the biological phase.

**Table S1.** Soil elements identified after digestion and ICP-OES analysis

| Element (ppb) | Blank | Sample |
| --- | --- | --- |
| Al | 282/2 | > 330382 |
| As | 1/3 | 130/0 |
| B | 227/2 | 1179/0 |
| Ba | 13/3 | > 1910.29 |
| Ca | 3821/4 | > 207374 |
| Cd | 1/7 | 7/9 |
| Cr | 177/58 | > 1548.45 |
| Cu | 20/1 | 459/9 |
| Fe | 579/0 | > 218285 |
| K | 253/5 | > 20064.1 |
| Li | 1/6 | 370/4 |
| Mg | 743/4 | > 202783 |
| Mn | 15/9 | > 10549.2 |
| Na | 19537/9 | 26775/9 |
| Ni | 77/3 | 766/3 |
| P | > 9410.69 | > 23087.2 |
| Pb | 33/1 | 1087/4 |
| Sr | 8/5 | 754/8 |
| V | < 0.064 | 534/0 |
| Zn | 483/4 | > 3782.42 |

**Table S2.** Dissolved oxygen measurement in both reactors

| DO – GAC Reactor | DO – Composite Reactor |
| --- | --- |
| 3/34 mg/L | 2/94 mg/L |
| 1/99 mg/L | 0/8 mg/L |
| 3/2 mg/L | 2/56 mg/L |

**Chart S1.** Violin plot showing the distribution and variability of pH values at the influent and effluent of GAC and Fe–GAC reactors during samples 16–40, representing the bioelectrochemical operational phase

**Figure S1.** GAC, and Fe-GAC reactors bacterial staining: Gram stain (a and b), acridine orange stain (c and d) with 100x magnification
